# EEG-based investigation of effects of mindfulness meditation training on state and trait by deep learning and traditional machine learning

**DOI:** 10.3389/fnhum.2023.1033420

**Published:** 2023-08-31

**Authors:** Baoxiang Shang, Feiyan Duan, Ruiqi Fu, Junling Gao, Hinhung Sik, Xianghong Meng, Chunqi Chang

**Affiliations:** ^1^School of Biomedical Engineering, Shenzhen University Medical School, Shenzhen University, Shenzhen, China; ^2^Department of Neurosurgery, Shenzhen University General Hospital, Shenzhen University, Shenzhen, China; ^3^Deepbay Innovation Technology Corporation Ltd., Shenzhen, China; ^4^Shenzhen Key Laboratory of Smart Healthcare Engineering, Southern University of Science and Technology, Shenzhen, China; ^5^Buddhist Practice and Counselling Science Lab, Centre of Buddhist Studies, The University of Hong Kong, Hong Kong, Hong Kong SAR, China

**Keywords:** meditation state classification, deep learning, electroencephalogram (EEG), state and trait characteristics, convolutional neural networks (CNN), mindfulness-based stress reduction (MBSR), filter bank common spatial pattern (FBCSP)

## Abstract

**Introduction:**

This study examines the state and trait effects of short-term mindfulness-based stress reduction (MBSR) training using convolutional neural networks (CNN) based deep learning methods and traditional machine learning methods, including shallow and deep ConvNets as well as support vector machine (SVM) with features extracted from common spatial pattern (CSP) and filter bank CSP (FBCSP).

**Methods:**

We investigated the electroencephalogram (EEG) measurements of 11 novice MBSR practitioners (6 males, 5 females; mean age 35.7 years; 7 Asians and 4 Caucasians) during resting and meditation at early and late training stages. The classifiers are trained and evaluated using inter-subject, mix-subject, intra-subject, and subject-transfer classification strategies, each according to a specific application scenario.

**Results:**

For MBSR state effect recognition, trait effect recognition using meditation EEG, and trait effect recognition using resting EEG, from shallow ConvNet classifier we get mix-subject/intra-subject classification accuracies superior to related previous studies for both novice and expert meditators with a variety of meditation types including yoga, Tibetan, and mindfulness, whereas from FBSCP + SVM classifier we get inter-subject classification accuracies of 68.50, 85.00, and 78.96%, respectively.

**Conclusion:**

Deep learning is superior for state effect recognition of novice meditators and slightly inferior but still comparable for both state and trait effects recognition of expert meditators when compared to the literatures. This study supports previous findings that short-term meditation training has EEG-recognizable state and trait effects.

## 1. Introduction

With the popularization of mindfulness meditation, especially the mindfulness-based stress reduction (MBSR) developed in a behavioral medicine environment suitable for people suffering from chronic pain, stress, depression and various other diseases (Khoury et al., 2015), this intrinsic neuromodulation method has gradually attracted widespread attention in the field of psychology and neuroscience. Since an 8 weeks short-term training of MBSR is effective on behavioral and brain function modulation even for novices (Kirk et al., 2016; Kral et al., 2018; Kwak et al., 2019; Favre et al., 2021; Rahrig et al., 2021; Guendelman et al., 2022), MBSR training is helpful for emotion and attention regulation, as well as decision making and executive functions, therefore has many clinical applications. It is important to assess the training effectiveness of MBSR practitioners and provide feedback to improve their performance (Farkhondeh Tale Navi et al., 2022; Yu et al., 2022). Therefore, mindfulness meditation state recognition is of great importance for online or offline feedback during MBSR training and practice (Brandmeyer and Delorme, 2020).

Neural mechanism of mindfulness meditation and neural characteristics of meditation state can be investigated by neuroimaging and neurophysiology using magnetic resonance imaging (MRI) (Gotink et al., 2016; Valk et al., 2017), functional magnetic resonance imaging (fMRI) (Engström et al., 2022; Li et al., 2022; Sezer et al., 2022; Snyder et al., 2022), functional near-infrared spectroscopy (fNIRs) (Bergen-Cico et al., 2021; Xie et al., 2022), electroencephalogram (EEG) (Ahani et al., 2014; Lomas et al., 2015; Ng et al., 2021; Wang et al., 2022), event-related potential (ERPs) (Gao et al., 2017; Lasaponara et al., 2019; Kaunhoven and Dorjee, 2021), magnetoencephalogram (MEG) (Berkovich-Ohana et al., 2013; Wong et al., 2015; Lardone et al., 2022), and various other biomedical engineering methods.

The most convenient method for characterizing meditation state is EEG, which is the physiological electrical activity of the brain recorded from the human scalp. Though short term mindfulness training may affect EEG functional connectivity (Xue et al., 2014; Travis, 2020; Trova et al., 2021), characteristics of mindfulness meditation can be more conveniently described in the spectral domain of EEG, especially in five standard frequency bands, namely delta band (1–4 Hz), theta band (5–8 Hz), alpha band (8–12 Hz), beta band (13–30 Hz), and gamma band (31–80 Hz), and reliable meditation characteristics have been found in theta and alpha bands (Cahn and Polich, 2006). In the meditation state, the theta rhythm in the frontal and temporal lobes is significantly stronger than in the occipital lobe. There is also a significant increase in amplitude and decrease in frequency of the posterior alpha rhythm at meditation states compared with resting condition (Lagopoulos et al., 2009). In addition, some studies have suggested that alpha regulation in the meditative state is a dynamic process: from amplitude increase to frequency decrease to alpha activity propagation in the frontal lobe, and finally the appearance of theta wave due to frequency decrease (Lee et al., 2018). Nonetheless, these results have not been consistently reported and no consistent patterns have been observed in the delta, beta and gamma bands in many EEG studies (Kerr et al., 2011; Ng et al., 2021; Śliwowski et al., 2021). Moreover, different meditation techniques and different aspects of the meditation may have their own specific EEG characteristics (Schoenberg and Vago, 2019).

Recognition of meditation state from EEG signal has gained some research interests, especially in the context of neurofeedback. Traditional machine learning techniques have been extensively applied for many different meditation styles using various feature extraction methods. Features used for meditation state recognition are from either frequency domain such as Fourier transform and time-frequency analysis, or spatial-temporal domain such as linear analysis using independent component analysis (ICA), common spatial patterns (CSP), and linear discriminator (LD), as well as non-linear analysis using entropy, correlation dimension (CD), largest Lyapunov exponent (LLE), and hurst exponent (HE) (Goshvarpour and Goshvarpour, 2012; Lin and Li, 2017; Han et al., 2020; Tee et al., 2020; Huang et al., 2021; Kora et al., 2021; Panachakel et al., 2021b). Brain connectivity features have also been exploited (Dissanayaka et al., 2015; Pandey et al., 2021). The classification techniques used for meditation state recognition include linear discriminant analysis (LDA) (Panachakel et al., 2021b), support vector machine (SVM) (Shaw and Routray, 2016; Han et al., 2020), random forest (RF) (Huang et al., 2021), and artificial neural network (ANN). Recently, deep learning techniques including long short-term memory (LSTM) framework (Panachakel et al., 2021a) as well as various deep convolutional neural networks (CNN) such as VGG16, ResNet50, and MobileNet (Pandey and Miyapuram, 2021), have also been exploited for meditation state recognition.

There are only a small number of publications reporting meditation state classification, mostly on yoga meditation (Han et al., 2020), including Raja yoga (Panachakel et al., 2021a,b), Kriya yoga (Shaw and Routray, 2016), and Himalayan yoga (Pandey and Miyapuram, 2021), with only one on non-specified meditation (Goshvarpour and Goshvarpour, 2012).

There is a still lack of research effort on meditation state classification during mindfulness meditation, especially on the well adopted MBSR training, and to the best of our knowledge only one publication is on brain state classification during mindfulness meditation which is not a standard 8-week MBSR but a 6-week program adapted from MBSR and mindfulness-based cognitive therapy (MBCT) (Ahani et al., 2014).

In this paper, we aim to perform EEG-based mindfulness meditation state classification during MBSR training, using deep learning methods as well as state-of-the-arts (SOTA) traditional machine learning approaches. Many deep learning and traditional machine learning methods have been applied to EEG-based brain state classification problems, as reviewed in Craik et al. (2019), Roy et al. (2019), Li et al. (2020), Gao et al. (2021), Saeidi et al. (2021), and Gong et al. (2022). While the popular deep learning architecture such as restricted Boltzmann machine (RBM), deep belief network (DBN), CNN, generative adversarial network (GAN), LSTM and gated recurrent unit (GRU) based recurrent neural networks (RNN), autoencoder (AE) and stacked AE (SAE), as well as some others such as capsule network (CapsNet), extreme learning machine (ELM), echo state network (ESN), Spiking neural network (SNN), and deep polynomial network (DPN), have all find applications for EEG analysis, those famous and effective deep network structures for audio, video, and image processing such as ImageNet, AlexNet, VGG, ResNet, and MobileNet are not suitable for most EEG applications, especially when only a small dataset is available.

Multi-channel EEG data are essentially spatio-temporal data, which have both the sampling of the two-dimensional surface of the brain scalp and the sampling over the time series, therefore EEG signals are different from images, sounds and video data. Moreover, EEG signals have information encoded in various oscillations, such as the delta, theta, alpha, beta and gamma rhythms. Two representative CNN deep learning networks developed specifically for EEG analysis are EEGNet (Lawhern et al., 2018) and deep/shallow ConvNet (Schirrmeister et al., 2017). The shallow ConvNet contains a CNN layer for spatial filtering and a dense layer for classification, and after the end-to-end training it conceptually trained a data-adaptive filter bank common spatial patterns (FBCSP) (Ang et al., 2012) for feature extraction and a following ANN for classification. The deep ConvNet is basically the same as shallow ConvNet but with a deep CNN instead of an ANN for classification, and they have been successfully applied for many brain state classification tasks such as depression recognition (Li et al., 2019), drowsiness recognition (Chen et al., 2021), and eye states classification (Han et al., 2022). Therefore, in this paper, we use deep and shallow ConvNets as the deep learning approaches and compare them with SVM classification (Shen et al., 2010; Dai et al., 2013, 2017) using CSP (Koles et al., 1990) and FBCSP as feature extraction methods.

Instead of a binary classification of meditation and resting as adopted in most meditation state classification literatures, in this paper we try also differentiate early and late stages of MBSR training for the aim of assessing the level of mindfulness meditation.

## 2. Materials and methods

### 2.1. EEG data

#### 2.1.1. Experimental procedure and EEG data collection

The EEG experiment was performed in the University of Hong Kong, and was approved by the Hong Kong Local Institutional Review Board (IRB). Eleven healthy participants volunteered to participate in the study (6 males, 5 females; mean age 35.7 years; 7 Asians and 4 Caucasians from local MBSR courses). All participants have a bachelor’s degree or above and had no previous experience in any kind of meditation before taking the MBSR training.

In this study, participants were taught mindfulness meditation in accordance with the standard MBSR training course which is an 8 weeks program with a maximum of 30 participants. The course generally includes 2–2.5 h group meeting each week for guided practice of mindfulness meditation and stress management techniques, 45 min daily homework, and a 1-day (7–8 h) retreat between week 6 and week 7. Three formal techniques including mindfulness meditation, body scanning and simple yoga postures, are instructed by the certified trainers.

**FIGURE 1:**
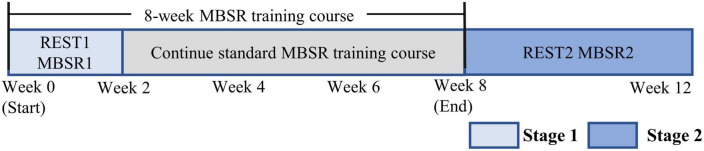
Flow chart of experimental design.

Mindfulness meditation states were investigated at two stages: the early stage (stage 1) after the beginning of the MBSR training (within 2 weeks, in weeks 1–2) and the late stage (stage 2) after the end of the training (within 4 weeks, in weeks 9–12). The experiments were performed in a quiet room, and at each stage, the participants were asked to do 10 min resting with eyes closed but do not think too much or fall asleep, with this period denoted as the resting state (REST1 and REST2 for stages 1 and 2, respectively), and then do 10 min mindfulness breathing taught in the MBSR course, with this period denoted as the mindfulness meditation state (MBSR1 and MBSR2 for stages 1 and 2, respectively), as shown in Figure 1. Scalp EEG data were recorded with a 128-channel Neuro-SCAN EEG system. More details about the experiment and data collection are described in Gao et al. (2016).

Two experiments were performed for each participant, with the first experiment in weeks 1–2, and the second experiment in weeks 9–12. Each experiment investigated two brain states: resting (denoted as REST1 and REST2, respectively) and mindfulness meditation (denoted as MBSR1 and MBSR2, respectively).

#### 2.1.2. EEG data pre-processing

The EEG data were preprocessed using the MATLAB toolkit EEGLAB (Delorme and Makeig, 2004) with the following steps before making brain state classification.

A number of 15 EEG channels (channels 10, 11, 17, 28, 59, 63, 64, 72, 74, 84, 85, 110, 111, 115, and 118) were excluded due to high impedance, with 113 EEG channels left for further processing and analysis. The remaining EEG data was resampled at the sampling rate of 250 Hz from the original sampling rate of 1,000 Hz, and then re-referenced to whole brain average reference from the original left mastoid reference. After that, notch filtering at 50 and 100 Hz was performed to reduce powerline noise, and 0.1–120 Hz bandpass filtering was followed to reduce low frequency signal drifting. At last, eye movement, eye blinking (EOG) and movement (EMG) artifacts were removed using the Automatic Artifact Removal (AAR) method (Gómez-Herrero, 2007) implemented in the EEGLAB toolkit.

After the pre-processing, for each participant, the EEG data were segmented into four segments according to the four brain states REST1, MBSR1, REST2, and MBSR2, respectively. Each segment is then divided into trials of 5 s, so that each brain state has 120 trials of data.

### 2.2. Brain state classification methods

#### 2.2.1. CSP + SVM

Support vector machine (SVM) is a robust and effective machine learning method which does not require very large dataset. Kernels can be used in SVM, with linear kernel for linear classification, and other kernels such as polynomial kernels and radial basis function (RBF) kernels for non-linear classification. Before applying SVM for brain state classification, EEG features rather than raw EEG data are preferred as the inputs to the classifier.

Common Spatial Pattern (CSP) analysis is a supervised spatial filtering method for multichannel EEG feature extraction. CSP applies basically to a two-classes classification problem and aims for both compression and discrimination. CSP analysis has mainly three steps. First get the covariance matrices for each group, denoted as C1 and C2, respectively, from the groups’ EEG data matrices E1 and E2. Then a whitening matrix is constructed from C = C1 + C2 and applied to C1 and C2 to get the whitened covariance matrices S1 and S2, which have identical eigen matrices U and their corresponding eigen value pairs summed to 1. Finally, the first two and the last two eigen vectors (corresponding to totally four filters) are chosen to project the EEG data matrices E1 and E2 to feature signal matrices F1 and F2, each having four rows of data. The logarithm of variance of each row of F1 and F2 is used as a feature, resulting in a feature vector of dimension 4, and then the feature vectors are used as inputs to the SVM classifier for training and classification. For multiple classes problem, we apply the one-vs.-rest (OVR) strategy for both CSP and SVM.

#### 2.2.2. FBCSP + SVM

Common spatial pattern gets discriminative features from the full-band raw EEG signals. Since as have been demonstrated in the literature, different frequency bands of the EEG signals may represent different brain functions and contribute differently to the characteristics of mindfulness meditation state, a temporal filter bank which decompose the EEG signals to a number of distinct frequency bands may be helpful before the EEG signals are projected by CSP spatial filters. After the EEG signals are decomposed into different frequency bands by the filter bank, each band of the signal is then utilized to obtain its corresponding CSP filter and subsequently its specific feature vectors, and at last all these feature vectors are combined to feed into the SVM for training and classification. The approach of filter bank plus CSP is denoted as Filter Bank Common Spatial Pattern (FBCSP). In this study, we construct a filter bank of 10 bandpass filters, each have a bandwidth of 4 Hz, i.e., 0–4, 4–8…36–40 Hz, to cover all the five EEG rhythms delta, theta, alpha, beta and gamma.

#### 2.2.3. Shallow ConvNet

The shallow ConvNet designed in Schirrmeister et al. (2017) is inspired from FBCSP. The filter bank in FBCSP is replaced by a temporal convolution layer in shallow ConvNet, and the following CSP spatial filter is replaced by a spatial convolution layer, and the SVM classifier is replaced by a mean pooling layer and fully connected dense layer of ANN. In the FBCSP + SVM framework, filter bank is manually designed, CSP spatial filters are mathematically designed according to the signal in each frequency band, and the SVM classifier is trained using the CSP extracted features. In the shallow ConvNet network, the whole network is trained end-to-end, so the filter bank and CSP are not deterministically specified but jointly optimized from the data. Theoretically, joint optimization is in general better than sub-problem optimization. The shallow ConvNet can be regarded as composed of feature extraction function and classifier function.

**FIGURE 2:**
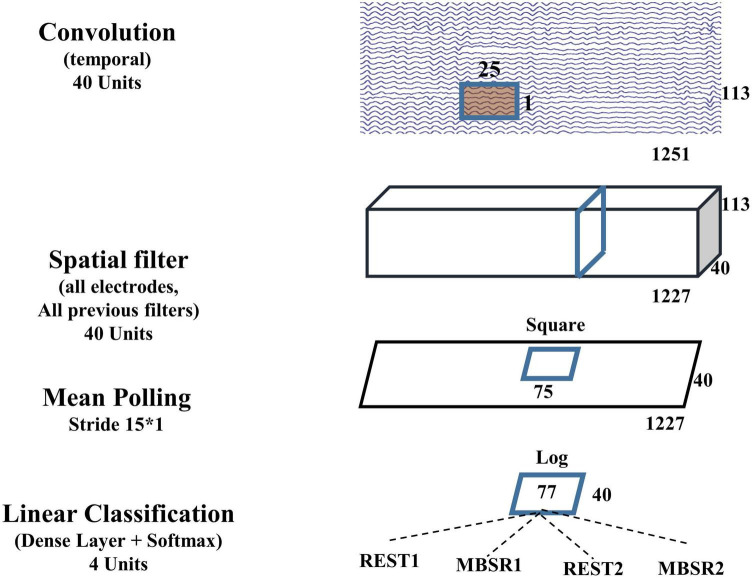
Shallow ConvNet.

In this study, the details of our shallow ConvNet adapted to our mindfulness meditation state classification problem are shown in Figure 2. In the temporal convolution layer, each trial of our EEG data has 113 channels (after bad channel removal) and 1,251 time points (5 s of data with a resampled sampling rate of 250 Hz), and we include 40 temporal filters in this layer to represent 40 filters in the filter bank. It should be noted that after the end-to-end training, these 40 filters are generally not non-overlapped bandpass filters but instead can be of any form. The spatial filter layer contains 40 spatial filters, corresponding to the 40 temporally filtering outputs. These spatial filters are expected to take the role of CSP spatial filters in FBCSP, but after end-to-end they may or may not get similar spatial filtering as those in FBCSP.

#### 2.2.4. Deep ConvNet

**FIGURE 3:**
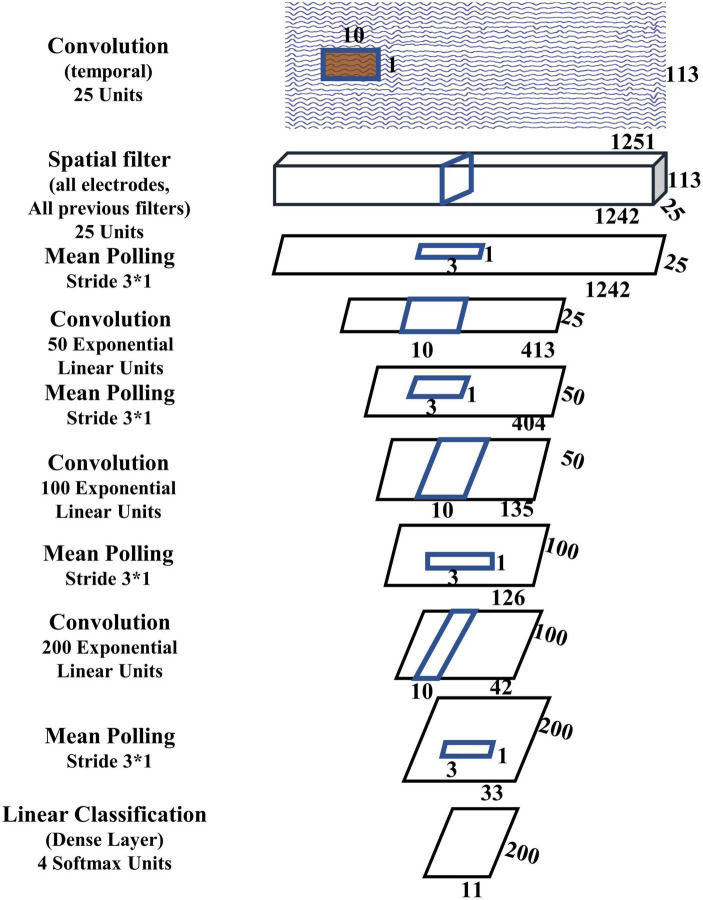
Deep ConvNet.

The shallow ConvNet uses a single layer ANN to replace the SVM classifier in the FBCSP + SVM framework. Since deep CNN architecture has been demonstrated in many applications to be superior than shallow architectures for large dataset, the ANN in shallow ConvNet can be replaced by deep CNN to form a deep ConvNet architecture (Schirrmeister et al., 2017), which inserts three convolution-max-pooling blocks between the spatial filter block and the final classification block. There are four convolutional pooling blocks in the deep ConvNets. The deep ConvNet used in this study is described in Figure 3, where we use 25 temporal filters in the first layer instead of 40 in the shallow ConvNet, with the main purpose of reducing network complexity.

### 2.3. Classification and training strategy

In addition to the four-class (MBSR1, REST1, MBSR2, and REST2) classification, we also performed five binary classifications including MBSR1/REST1, MBSR2/REST2, and MBSR/REST for mindfulness/rest brain states classification at the two stages and their combination, as well as MBSR1/MBSR2 and REST1/REST2 for stage classification corresponding to mindfulness state and resting state, respectively. The class MBSR combines MBSR1 and MBSR2, and the class REST combine REST1 and REST2.

Since we have EEG data from a group of subjects, how to arrange training and classification across subject should be considered (Kamrud et al., 2021). In this study, four scenarios are investigated, including inter-subject classification, individual-subject classification, intra-subject classification, and transfer learning. For each scenario, we choose randomly 60% of the EEG trials as training set, 20% as validation set, and 20% as testing set, for deep and shallow ConvNets, while we use 80% as training and 20% as testing for CSP/FBCSP + SVM. We repeat this data-dividing and classification for 11 times to get an average performance. For the inter-subject classification scenario, leave-one-subject-out cross validation (LOOCV) is performed.

#### 2.3.1. Inter-subject classification

In order to be applicable to the general population whose EEG data are not available during the training of the classifier, the classifier should be trained on a specific group of training subjects and then applies to the general population. This training strategy is called inter-subject or cross-subject training. For inter-subject training/classification, the leave-one-subject-out cross validation (LOOCV) method was adopted in this study. Among the EEG data of 11 subjects, each of them was left in turn for testing and the data of the other 10 subjects are used for training, where 80% trials of the training subjects are used as training set and 20% trials were used as validation set for the training of deep and shallow ConvNets, whereas for CSP/FBCSP + SVM all EEG trials of the training subjects are used for training. Therefore, for each classification method, we have 11 trained classifiers, each for a specific testing subject. The principle of LOOCV is to guarantee that the subject used for testing cannot be mixed into the training set, so no data leakage occurs during LOOCV.

#### 2.3.2. Mix-subject classification

In this so-called mix-subject classification scenario, we mix data from all subjects and divide the EEG trials as training, validation (for deep and shallow ConvNets) and testing set randomly. This classification strategy is user-independent, as pointed out by Kamrud et al. (2021), if an inter-subject model is intended to perform classification on only the same population where the training subjects are drawn, but not also unseen individuals, then testing the model on unseen subjects is unnecessary. This user-independent mix-subject classification strategy has been adopted by most previous studies for meditation state classification (Ahani et al., 2014; Shaw and Routray, 2016; Han et al., 2020).

#### 2.3.3. Intra-subject classification

The purpose of both inter-subject and mix-subject classification strategies are for effective applications in unseen (by the training) subjects from the general population and the population same as the training subjects, respectively. However, inter-subject classification performs usually much poorer than mix-subject classification (Kamrud et al., 2021; Fu et al., 2022). If the mix-subject classification strategy is not applicable due to population shift and the inter-subject classification performs not good enough, the alternative intra-subject or within-subject classification strategy is to collect some EEG data from the target subject and use such EEG data for training and testing, and the classifier trained this way can be used by this specific subject for future mindfulness state recognition applications such as neurofeedback assisted mindfulness training. The advantage of the intra-subject classification strategy is that the classifier is dedicatedly trained for the specific target subject so does not suffer from the problem caused by individual differences, while its disadvantage is that the classifier is not readily available from the beginning because some training data need to be collected in advance and the classifier should be trained again from these training data. Although intra-subject classification may have some narrowly limited application scenario as discussed above for meditation states classification, it can hardly have any practical application for meditation experience classification. This strategy is studied only for state classification in this paper.

#### 2.3.4. Subject-transfer classification

Another disadvantage of the intra-subject classification strategy is that in general the classifier can only be trained from a relatively small dataset from a single subject and limited time span. For deep learning methods, especially those with very deep structures and thus a huge amount of model parameters to be learned from the data, a relatively large dataset is necessary for obtaining an effective model. To overcome this shortage of data problem, the idea of transfer learning (Pan and Yang, 2010; Niu et al., 2020) can be applied, and specifically we adopt a subject transfer strategy (Samek et al., 2013; Zhao et al., 2019). In this subject-transfer classification strategy, the inter-subject classification and intra-subject classification strategies are combined. For each target subject, we first train a subject-independent classifier using the same procedure as described in the inter-subject classification strategy, and then we finetune this classifier using the EEG data of the target subject and get a subject-transferred classifier. In the finetuning, the division of the EEG data of the target subject into training, validation, and testing sets follows the same as in the intra-subject classification strategy. In this study this subject-transfer classification strategy applies only to deep and shallow ConvNets but not CSP and FBCSP assisted SVM, since only deep networks can be trained by finetuning (Pan and Yang, 2010; Niu et al., 2020). For the same reason as in intra-subject classification, the subject-transfer classification strategy is also studied only for state classification.

## 3. Results

For each of the four classification strategies (inter-subject, mix-subject, intra-subject, and subject-transfer), each of the four classification methods (CSP + SVM, FBCSP + SVM, shallow ConvNet, and deep ConvNet), and each of the six classification tasks (MBSR1/REST1/MBSR2/REST2, MBRS1/REST1, MBSR2/REST2, MBSR/REST, MBSR1/MBSR2, and REST1/REST2), we perform either 11 times of LOOCV or 11 times of training/classification with random division of the data, and then get the average classification accuracy for each combination of classification strategy and classification method. The results are then grouped according to the classification strategy and presented in the form of both tables and figures. Statistical tests on the performance of the two deep learning methods (deep and shallow ConvNets) using the non-parametric Mann-Whitney *U*-test of two independent samples (Nachar, 2008) and on six different classification tasks using k-independent sample Kruskal-Wallis test (Vargha and Delaney, 1998), are also presented for each classification strategy if applicable.

### 3.1. Inter-subject classification

**FIGURE 4:**
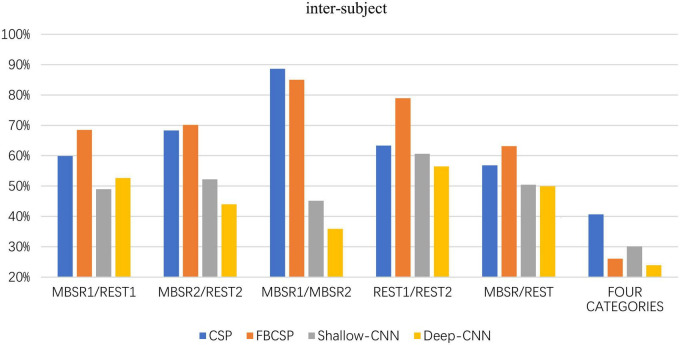
Classification accuracies for inter-subject classification.

The classification accuracies are presented in Table 1 and also in Figure 4. The non-parametric Mann-Whitney *U*-test of two independent samples finds that the difference of classification accuracy between the deep and shallow ConvNets is not significant (*Z* = −0.665, *p* = 0.512), but the k-independent sample Kruskal-Wallis test shows that the difference among the six categories is significant (*Z* = 34.344, *p* = 0.000).

**TABLE 1:** Classification accuracies for inter-subject classification.

	Classification task	Shallow ConvNet	Deep ConvNet	CSP	FBCSP
State	MBSR1/REST1	48.95%	52.65%	59.93%	68.49%
	MBSR2/REST2	52.24%	44.02%	68.31%	70.17%
Stage	MBSR1/MBSR2	45.14%	35.90%	88.66%	85.00%
	REST1/REST2	60.66%	56.46%	63.35%	78.96%
Combined	MBSR/REST	50.42%	49.96%	56.85	63.17%
4-class	All four classes	30.12%	23.92%	40.69%	26.09%

### 3.2. Mix-subject classification

**FIGURE 5:**
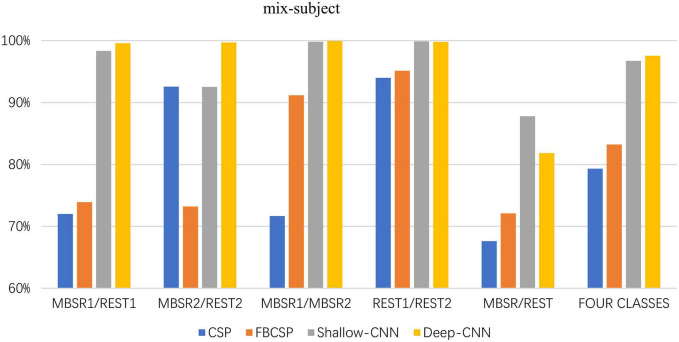
Classification accuracies for mix-subject classification.

The classification accuracies are presented in Table 2 and also in Figure 5. We find that the classification accuracies between the two convolutional neural networks are significantly different (*Z* = 2.795, *p* = 0.005) using the non-parametric Mann-Whitney *U*-test. The classification accuracy of deep ConvNet is significantly better than that of the shallow ConvNet. The non-parametric k-independent sample Kruskal-Wallis test finds that the results of the six classification tasks are significantly different (*Z* = 98.650, *p* = 0.000).

**TABLE 2:** Classification accuracies for mix-subject classification.

	Classification task	Shallow ConvNet	Deep ConvNet	CSP	FBCSP
State	MBSR1/REST1	98.33%	99.59%	72.01%	73.92%
	MBSR2/REST2	92.53%	99.70%	92.56%	73.23%
Stage	MBSR1/MBSR2	99.81%	99.95%	71.67%	91.16%
	REST1/REST2	99.88%	99.78%	93.98%	95.13%
Combined	MBSR/REST	87.79%	81.83%	67.63%	72.10%
4-class	All four classes	96.72%	97.53%	79.32%	83.23%

### 3.3. Intra-subject classification

The classification accuracies are presented in Table 3.

**TABLE 3:** Classification accuracies for intra-subject classification.

	Classification task	Shallow ConvNet	Deep ConvNet	CSP	FBCSP
State	MBSR1/REST1	99.01%	97.05%	82.50%	94.60%
	MBSR2/REST2	97.99%	83.85%	80.24%	94.83%
Combined	MBSR/REST	99.26%	98.60%	85.12	87.40%

### 3.4. Subject-transfer classification

The classification accuracies are presented in Table 4.

**TABLE 4:** Classification accuracies for subject-transfer classification.

	Classification task	Shallow ConvNet	Deep ConvNet
State	MBSR1/REST1	99.64%	96.84%
	MBSR2/REST2	98.06%	95.15%
Combined	MBSR/REST	99.05%	98.29%

## 4. Discussion

### 4.1. Comparison with the literatures

#### 4.1.1. State classification

In the literature there are four published studies on classifying meditation states and resting states of the same subject using EEG measurements, as detailed in Table 5, where the results of our study on meditation and resting state classification, represented by the average accuracy of the two stages, i.e., MBSR1/REST1 and MBSR2/REST2, are also presented for ease of comparison.

**TABLE 5:** Comparison to the literatures on mediation state classification; the accuracy of our methods on meditation/resting classification is the average for MBSR1/REST1 and MBSR2/REST2; SCNN and DCNN represent shallow and deep ConvNets, respectively.

References	Meditation	Tasks	Experience	Strategy	Method	Accuracy
Panachakel et al., 2021a	Raja yoga	Med/rest	Expert	Inter-subj	LSTM-α	79.1%
					β	86.5%
					Low γ	91.0%
					High γ	94.1%
Panachakel et al., 2021b	Raja yoga	Med/rest	Expert	Inter-subj	LDA	74.0%
				Intra-subj		97.9%
Han et al., 2020	Yoga	Med/rest/attention	Expert	Mix-subj	SVM	74.31%
			Novice			62.16%
Ahani et al., 2014	Mindfulness	Med/rest	Novice	Mix-subj	SVM	78%
Ours	MBSR	Med/rest	Novice	Inter-subj	SCNN	50.60%
					DCNN	48.34%
					FBCSP	68.50%
					CSP	64.12%
				Mix-subj	SCNN	95.43%
					DCNN	99.65%
					FBCSP	73.58%
					CSP	82.29%
				Intra-subj	SCNN	98.40%
					DCNN	90.45%
					FBCSP	94.72%
					CSP	81.37%
				Transfer	SCNN	98.85%
					DCNN	96.00%

Mix-subject classification of mindfulness meditation state for a group of novice meditators participating in a short-term MBSR/MBCT adapted mindfulness training program is presented in Ahani et al. (2014), where SVM is used to obtain a classification accuracy of 78%, which is very close to our CSP and FBCSP based results (82.29 and 73.58%, respectively, with mean value of 77.94%), but is much inferior to the performance of our deep and shallow ConvNets (with classification accuracy of 99.65 and 95.43%, respectively).

Meditation state classification for expert and novice yoga meditators using SVM is reported in Han et al. (2020) with the accuracy of 74.31 and 62.16%, respectively, according to a mix-subject classification strategy, where it demonstrates that the discrimination between meditation and resting is much more difficult for novice than for expert meditators, since the meditation expertise of the novices is much lower than that of experts.

In the intra-subject classification scenario, using the traditional machine learning technique CSP + LDA, for Raja yoga experts, (Panachakel et al., 2021b) reports a classification accuracy of 97.9%, very much higher than our result (82.29%) using similar machine learning technique (CSP + SVM) but for MBSR novices. This implies that the task of meditation state recognition in the settings of our study for novice MBSR practitioners is much more difficult and challenging than that for Raja yoga experts. However, for the same intra-subject scenario for MBSR novices in our study, both shallow and deep ConvNets obtain very promising and improved classification accuracy, which are 98.40 and 90.45%, respectively, and the subject transfer learning further improves the accuracy to 98.80 and 96.00%, respectively.

The inter-subject classification accuracy using traditional machine learning technique FBCSP + SVM in our study is 68.50%, which is comparable to 74.0% reported in Panachakel et al. (2021b) using also traditional machine learning method, but as discussed above our task is much more difficult. The inter-subject classification accuracy for Raja yoga experts is greatly improved from 74.0% in Panachakel et al. (2021b) to 79.1, 86.5, 91.0, and 94.1% in Panachakel et al. (2021a) for using alpha, beta, low gamma, high gamma features, respectively, followed by the CSP + LDA + LSTM deep learning framework. However, the meditation state classification accuracy for our MBSR novices using deep and shallow ConvNets does not improve over the traditional FBCSP + SVM method but instead drops to around chance levels of 48.34 and 50.60%, respectively. Though in this study we use a CNN architecture ConvNet as the deep learning architecture, which is different from the RNN architecture LSTM in Panachakel et al. (2021a), the main reason accounting for the failure of ConvNet in the inter-subject classification scenario should be that ConvNet uses an end-to-end training architecture whereas in Panachakel et al. (2021a) the LSTM architecture does not work directly on raw EEG data but instead on EEG features extracted by CSP + LDA.

#### 4.1.2. Stage classification

Meditation expertise increases through training and practice, and there will be both state and trait effects which can be characterized in either behavioral data or brain activities such as resting EEG for trait effect and meditating EEG for state effect (Cahn and Polich, 2006; Zarka et al., 2022). There is lack of studies on classification of brain states for different training and practicing stages longitudinally for individual meditation practitioners, but some efforts have been spent on classifying subjects with different levels of meditation expertise using EEG in either the meditating states (Shaw and Routray, 2016; Lee et al., 2017; Pandey and Miyapuram, 2021) or the resting states (Sharma et al., 2019), as detailed in Table 6, where the results of our study in this paper on meditation stage classification using meditation EEG (MBSR1/MBSR2) and resting state EEG (REST1/REST2), are also presented for ease of comparison.

**TABLE 6:** Comparison to the literatures on mediation experience/expertise classification using meditation and resting EEG; in our study, meditation EEG and resting EEG based classifications are corresponding to MBSR1/MBSR2 and REST1/REST2, respectively; SCNN and DCNN represent shallow and deep ConvNets, respectively.

References	Meditation	Tasks	Strategy	Method	Accuracy	
					Meditation	Resting
Pandey and Miyapuram, 2021	Himalayan Yoga	Expert/non-expert/non-meditator (S/J/N)	Inter-subj	VGG16	97.27%	
				ResNet50	91.01%	
				MobNet	90.57%	
				MobNet2	88.73%	
				LightCNN	94.57%	
Lee et al., 2017	Tibetan	S/J/N	Mix-subj	ANN	99.05%	
Shaw and Routray, 2016	Kriya Yoga	Expert/novice	Mix-subj	SVM	85.54%	
				k-SVM	90.83%	
Sharma et al., 2019	Yoga and SK	Train/control	Mix-subj	ANN		87.2%
Ours	MBSR	Stage 1/Stage 2	Inter-subj	SCNN	45.14%	60.66%
				DCNN	35.90%	56.46%
				FBCSP	85.00%	78.96%
				CSP	88.66%	63.35%
			Mix-subj	SCNN	99.81%	99.88%
				DCNN	99.95%	99.78%
				FBCSP	91.16%	95.13%
				CSP	71.67%	93.98%

In Pandey and Miyapuram (2021), expert and non-expert Himalayan yoga meditators, along with non-meditator healthy controls are classified by their meditation experience/expertise according to the EEG data measured when they are asked to do a focused-attention (to breath sensations) meditation, using a variety of a variety of CNN deep learning architectures including VGG16, ResNet50, MobileNet, MobileNet-2, and a lightweight CNN, with high inter-subject classification accuracy of 97.27, 91.01, 90.57, 88.73, and 94.57%, respectively. The two stages of MBSR training/practicing in our study are chosen as Weeks 1–2 and Weeks 9–12 after the starting of MBSR training, for which the meditation expertise of the subject is relatively higher in stage 2 than in stage 1, but reasonably far not as discriminable as among experts, non-experts, and non-meditators. Nevertheless, our traditional machine learning method FBCSP + SVM, and the deep learning methods shallow and deep ConvNets for MBSR1/MBSR2 classification all perform almost perfectly on non-inter-subject classification scenarios, with respective classification accuracy as 91.16, 99.81, and 99.95% for mix-subject classification. As a comparison, in the mix-subject classification scenario, the classification of senior/junior/novice Tibetan Nyingmapa meditation expertise attains an average accuracy of 99.05% as reported in Lee et al. (2017), and the classification of expert/novice Kriya yoga meditation experience using SVM and kernel SVM (k-SVM) has classification accuracy of 85.54 and 90.83%, respectively, as reported in Shaw and Routray (2016). However, for inter-subject classification, the two deep learning ConvNets do not perform satisfactorily, while the traditional machine learning methods CSP + SVM and FBCSP + SVM still perform reasonably well with classification accuracy of 88.66 and 85.00%, respectively, comparable to 91.01, 90.57, and 88.73% reported in Pandey and Miyapuram (2021) for a theoretically more discriminative task of experts/non-experts/non-meditators classification.

For EEG-based classification of meditation experience using trait characteristics, in Sharma et al. (2019) an ANN is designed to recognize combined Yoga and Sudarshan Kriya meditation experience from resting state EEG data and its mix-subject classification accuracy is 87.2%, which is much inferior to the resting state EEG based stage classification performance for MBSR practitioners, where the REST1/RESR2 mix-subject classification accuracy is 93.98, 95.13, 99.88, and 99.78% for the classification methods CSP, FBCSP, shallow ConvNet, and deep ConvNet, respectively. However, as expected, since trait characteristics are not as discriminative as state characteristics, inter-subject classification of REST1/REST2 by FBCSP can attain the accuracy of only 78.96%, lower than 85.00% in the case of using meditation EEG.

### 4.2. Classification tasks

In addition to binary classifications of meditation states from resting states at each of the two stages and their combinations as well as stage combination using meditation and resting EEG, we also perform a multi-class classification on all the four different brain states across the two training stages. Both the traditional machine learning method FBCSP + SVM and the deep learning method shallow ConvNet perform quite well for mix-subject and intra-subject classification scenarios, demonstrating that there are both state and trait effects for the short-term MBSR training and such two kinds of effects are also discriminable from each other. Since no such longitudinal combined trait and state meditation recognition has ever been investigated in any previous research project, we could not find a proper previous study for comparison. One related study is a three-class (meditation/rest/attention) classification reported in Han et al. (2020) where the mix-subject classification accuracy is 74.31% for experts and 62.16% for novices, whereas in our study of novice MBSR practitioners, the four-class (MBSR1/REST1/MBSR2/REST2) mix-subject classification accuracy is 83.23% using FBCSP and 96.72% using shallow ConvNet, and this demonstrates the effectiveness of both our MBSR training program and our classification methods.

As shown in Tables 1–4, in total we have six classification tasks in this study, and statistical tests show that for each and all of the four classification scenarios, difference in classification accuracy among the six tasks is significant. This difference happens in part by the fact that the two classification tasks MBSR/REST and MBSR1/REST1/MBSR2/REST2 are more difficult than the other four binary classification tasks, and in part by the fact that meditation state is more discriminable from resting state at Stage 2 than at Stage 1 (MBSR1/REST1 is more difficult than MBSR2/REST2) and state meditation characteristics is more discriminative than trait meditation characteristics (REST1/REST2 is more difficult than MBSR1/MBSR2), which is well demonstrated by the inter-subject classification results of the CSP and FBCSP methods shown in Table 1.

Across all the four classification scenarios and all the four classification methods under investigation, there is a general and consistent trend that stage tasks (MBSR1/MBSR2 and REST1/REST2) are classified with higher accuracy than the state tasks (MBSR1/REST1 and MBSR2/REST2). This may imply that the short-term MBSR training is very effective that the brain functional networks have been greatly modulated to produce significantly different EEG characteristics in both the meditation and the resting state, as compared to the pre-training period. However, the EEG differences between the two stages may also be caused by some other meditation unrelated changes in either measurement or cognition environment that affect the EEG features. This may be investigated further in some future research.

### 4.3. Classification methods

#### 4.3.1. Deep learning vs. traditional machine learning

For relatively larger dataset, in the mix-subject classification scenario, both shallow and deep ConvNets outperform greatly CSP and FBCSP, and for small dataset in the intra-subject classification scenario, the performance of shallow ConvNet is even more outstanding while the deep ConvNet performs comparably to FBCSB and better than CSP. However, for inter-subject classification, the pre-trained shallow and deep ConvNets fail to generalize to unseen subjects, while the performance of CSP and FBCSP work still reasonably well.

Within the two traditional machine learning methods, for all the three classification scenarios, FBCSP in general have overall performance better than CSP. This is expected since FBCSP extract EEG rhythms which contribute to the neuro-mechanisms of meditation so can better discriminate meditation states from resting states, while CSP mixes these rhythms into a wideband continuous signal so may not be as discriminative as FBCSP.

As for the two deep learning methods, their performance depends on the size of data available for model training. The more complex the network, the more data are needed. On one hand, for intra-subject, statistical test on the performance of deep ConvNet over shallow ConvNet find the statistics *Z* = −1.923 which is marginally significant (*p* = 0.05), where the negative Z shows the shallow ConvNet performs better than deep ConvNet. A possible reason is that for intra-subject classification we have training EEG data only from a single subject with a sample size not big enough for deep ConvNets to achieve sufficient optimization for better classification than shallow ConvNet which is simpler and requires less data for effective training, and this is also demonstrated by the subject-transfer learning where the classification accuracy for deep ConvNet is slightly but consistently improved for almost all the six classification tasks due to the additional data used for pre-training the network. On the other hand, for mix-subject classification, the statistical test gets *Z* = 2.795 and *p* = 0.005, which means deep ConvNet is significantly better than shallow ConvNet. It may be because the sample size in the mix-subject scenario is large enough for the more complex deep ConvNet to get sufficiently optimized to outperform the shallow ConvNet. Moreover, for inter-subject and subject-transfer classification, the statistical test gets *Z* = −0.665 (*p* = 0.512) and −1.590 (*p* = 0.112), respectively, meaning in such two cases the shallow ConvNet performs better but not to the level of statistical significance, implying that more data are needed in order to improve inter-subject classification using either shallow ConvNet or deep ConvNet.

#### 4.3.2. End-to-end learning tries to catch subject-dependent features

The deep learning ConvNet architecture, especially the shallow ConvNet, performs much better than the traditional machine learning methods CSP and FBCSP for both mix-subject classification and intra-subject classification, where in the latter we have very few EEG samples for training the networks. This good performance is due to the extreme simplicity of shallow ConvNet which mimics a very simple FBCSP + ANN architecture with a small number of model parameters. Different from a two steps approach of first FBCSP and then ANN where each step is trained separately, the two blocks in the shallow ConvNet are jointly optimized through an end-to-end learning strategy. In the deep ConvNet, the single layer ANN in the shallow ConvNet is replaced by a deep CNN architecture with three more CNN-max-pooling blocks, and the whole network is also jointly optimized through end-to-end learning.

The better performance of shallow ConvNet than that of FBCSP in the intra-subject classification scenario is definitely due to this joint optimization, and better performance of deep ConvNet than shallow ConvNet in the mix-subject classification scenario is due to the availability of larger data that makes a deeper architecture get more discriminative features. However, the end-to-end joint optimization is a mixed blessing, which on the one hand gets very individualized subject-dependent features so as to attain superior intra-subject and mix-subject classification performance for that specific subject or population, but on the other hand is difficult to generalize to unseen subjects with big individual difference. This is why deep and shallow ConvNets perform badly in our study for inter-subject classification while FBCSP still has reasonably good performance.

To overcome this disadvantage of end-to-end ConvNets on inter-subject classification, we may either enlarge the dataset by collecting data from a large number of subjects for better covering broad individual features so as to reduce the problem of individual difference, or try not to use end-to-end learning for the case where only a small number of subjects are available, as demonstrated in Panachakel et al. (2021a).

### 4.4. Classification strategies

The performance of mindfulness meditation state classification by deep learning methods deep and shallow ConvNets as well as traditional machine learning methods CSP and FBCSP is evaluated in four different application scenarios, each having its own practical applications on either meditation level evaluation or meditation training through neurofeedback.

For intra-subject classification, shallow ConvNet attains excellent classification performance with classification accuracy not less than 98.0% for all the five binary classification tasks, while the performance of both deep ConvNet and FBCSP is also quite promising. The intra-subject classification strategy requires the target subject to perform the whole MBSR training program first in order to use the EEG data collected at the two stages to train the meditation state classifiers. Though the trained classifier can be used to assist the practice of MBSR for the target subject subsequently, it cannot be used at the beginning of the MBSR training. In addition, the performance of subsequent application of the same target subject may still cannot be guaranteed despite the excellent intra-subject classification performance, since there may be inter-session variability that might significantly degrade or corrupt the classification ability for data from new sessions, similar to the degradation in the inter-subject classification scenario demonstrated in this study.

The mix-subject classification strategy relaxes the requirement to collect data from the target subject to data from a relatively homogeneous population covering the target subject. If this homogeneous population requirement is fulfilled, then a classifier trained from EEG data of a subset of the population can be evaluated on the training subject and applies directly to the unseen target subject from the same population, expecting similar performance as already evaluated in the mix-subject setting. The mix-subject classification performance in our study is also quite good for deep and shallow ConvNets, and therefore if we do have such kind of homogeneous population for application, then the classifier trained this way may generalize well for unseen target subjects. As a matter of fact, in our study, for inter-subject classification, which can be regarded as an application of mix-subject classification to unseen subjects, performs poorly for the ConvNets as well as significantly inferior to mix-subject classification for CSP and FBCSP. This implies that the subjects in our study are far from homogeneous, which is true since for the 11 subjects in our study we have 6 males and 5 females, with 7 of them are Asians and 4 Caucasians.

If collecting in advance the EEG data from the target subject is either inconvenient or impractical and the requirement for mix-subject classification cannot be fulfilled, then in order to get a good classifier generalizable to unseen target subjects, the classifier should be trained and evaluated using the inter-subject classification strategy. Though in our study deep and shallow ConvNets fail for inter-subject classification, the performance of FBCSP is reasonably good for both state and stage classification tasks, especially when considering that our tasks of meditation state classification for short-term training of novices are considerably more difficult than what is reported in the literatures where expert meditators are involved and compared to non-meditator controls.

For deep learning networks, if data available for model training is not sufficiently large to guarantee effective parameters optimization, then transfer learning may get more data to improve the training. Data shortage may be a problem of intra-subject classification since only a single subject is used for training. Though both shallow and deep ConvNets in our study perform excellently for intra-subject classification since they are relatively much simpler than those popular models such as VGG16 and ResNet50, more complex deep learning models may have degraded performance due to data shortage in the intra-subject setting, and then subject-transfer learning may help improve the intra-subject classification performance. In our study, most of the intra-subject classification accuracies of deep ConvNet for the six classification tasks are indeed significantly elevated by subject-transfer learning. Subject-transfer learning may also help inter-subject classification when we use only a small part of the unlabeled EEG data of the target subject to finetune the classifier so as to reduce the effect of inter-subject variability as demonstrated in for applications using speech and electrocardiogram (ECG) signals (Xu et al., 2022).

For meditation state/experience classification, the intra-subject classification strategy has been used in Panachakel et al. (2021b), the mix-subject classification strategy has been used in Ahani et al. (2014), Khoury et al. (2015), Shaw and Routray (2016), Lee et al. (2017), Sharma et al. (2019), and Han et al. (2020), and the inter-subject classification strategy has been used in Panachakel et al. (2021a,b) and Pandey and Miyapuram (2021). The inter-subject classification strategy is most suitable for the general case but it is the most difficult due to inter-subject variation. Mix-subject classification may avoid the over-fitting problem of intra-subject classification with the use of more data for training, and it may closely approximate the inter-subject classification if the subjects are from a homogenous population. In the case of this paper, the 11 subjects are quite non-homogeneous, therefore the inter-subject classification accuracies are much lower than those of mix-subject classification. Moreover, since the early and late stage of mindfulness meditation may be different for individual subjects, that is to say, the time accumulation effect of meditation may not be the same for all subjects, making the classification of meditation experience more challenging. Though intra-subject classification may have some narrowly limited application scenario as discussed above for meditation states classification, it can hardly have any practical application for meditation experience classification. Finally, the subject-transfer classification strategy combines both intra-subject and mix-subject classification strategies in order to overcome the limitations of each of the two individual strategies.

### 4.5. Feature analysis

**FIGURE 6:**
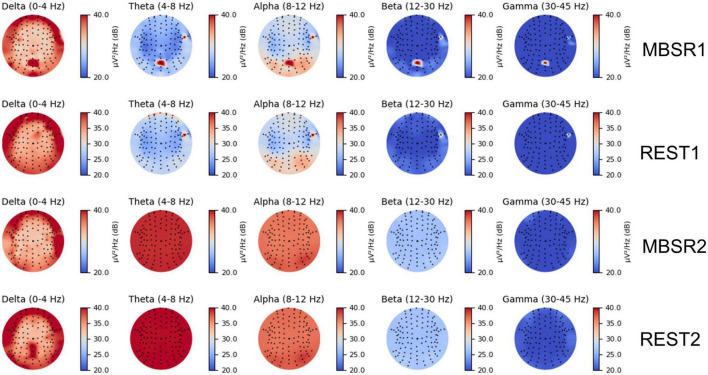
Power variation network-prediction correlation topographies of five frequency bands for deep ConvNet at the four different mental states.

To demonstrate that the CNN network is reliable for identifying meditative and resting EEG features, we visualized PSD topologies of raw EEG data in Figure 6 and reviewed the relevant literature. By referring to the results of traditional analysis methods and previous conclusions, we conduct correlation analysis between CNN network features and PSD features.

Region of Interest (ROI) were defined with the occipital lobe (channels N19, N20, N21, N41, N42, N43, N44, N45, N46, N67, N68, N69, N70, N71, N72, N96, N97, N98, N99, and N100), the middle frontal lobe (channel N53, N54, N55, N56, N57, N58, N59, N60, N61, N79, N80, N81, N82, and N83) and the middle parietal lobe (channels N48, N49, N50, N64, N65, N66, N74, N75, and N76). The selected EEG channels have been proved to be mindfulness-related (Gao et al., 2016). Alpha waves (8–12 Hz) and beta waves (12–30 Hz) are enhanced and delta waves (1–4 Hz) are decreased during mindfulness-based stress reduction exercises compared to resting states. The increase in alpha waves was significant throughout the brain, especially in the frontal and occipital lobes. The increase in beta waves is mainly concentrated in the frontal lobe. Delta waves are reduced in the centro-parietal region.

For analysis of the features learned by the CNN models and to illustrate that these features are mindfulness-related, the canonical correlation analysis (CCA) (Hardoon et al., 2004) between two feature groups including CNN-based and handcraft-based features were conducted. CNN-based features are extracted by the two CNN models from the data of each subject (also mixed subjects) at each MBSR and REST tasks, they are the output of the layer before the classification layer of the CNN networks. Handcraft-based features include the ratio of PSD value between delta and alpha bands of certain channels, also the ratio of PSD value between delta and beta bands of region of interest of the recorded EEG data.

The *r*-value of CCA between the CNN-based features and each sub-feature set in handcraft-based features is shown in Table 7, including two sub-tables for deep and shallow ConvNet, respectively. The first eleven columns demonstrate the correlation and significance between network feature of each subject at each task and one of the sub-feature sets of the handcraft-based features, the last column shows the correlation and significance between network feature of mixed subjects at each task and the handcraft-based features. In Table 7, the chance level is the *r*-value between CNN-based features and random white noise.

**TABLE 7:** Correlation values (*r*) of canonical correlation analysis (CCA) between deep and shallow ConvNet derived features and handcraft-based features (band power ratios δ/α and δ/β) for each subject (also mixed subjects) at the four mental states, with * indicates significant correlation (*p* < 0.05) when compared to the corresponding chance level correlations.

Deep		1	2	3	4	5	6	7	8	9	10	11	Ave	Mix
MBSR1	δ/α	0.91*	0.82*	0.82*	0.82*	0.76*	0.77*	0.85*	0.83*	0.74*	0.8*	0.81*	0.81 ± 0.05	0.76*
	δ/β	0.88*	0.82*	0.84*	0.83*	0.8*	0.76*	0.81*	0.78*	0.88*	0.8*	0.85*	0.82 ± 0.04	0.75*
	Chance	0.14 ± 0.02	0.34 ± 0.01	0.12 ± 0.02	0.38 ± 0.01	0.35 ± 0.01	0.31 ± 0.01	0.12 ± 0.02	0.17 ± 0.02	0.38 ± 0.01	0.18 ± 0.01	0.12 ± 0.02	0.24 ± 0.11	0.24 ± 0.01
MBSR2	δ/α	0.97*	0.94*	0.94*	0.98*	0.98*	0.95*	0.77*	0.94*	0.81*	0.98*	0.99*	0.93 ± 0.07	0.95*
	δ/β	0.97*	0.98*	0.91*	0.97*	0.98*	0.92*	0.92*	0.94*	0.85*	0.98*	0.99*	0.95 ± 0.04	0.95*
	Chance	0.17 ± 0.01	0.26 ± 0.01	0.18 ± 0.02	0.15 ± 0.01	0.19 ± 0.01	0.34 ± 0.01	0.35 ± 0.01	0.14 ± 0.02	0.26 ± 0.01	0.23 ± 0.01	0.19 ± 0.01	0.22 ± 0.07	0.28 ± 0.01
REST1	δ/α	0.66*	0.68*	0.61*	0.67*	0.64*	0.66*	0.57*	0.54*	0.64*	0.61*	0.56*	0.62 ± 0.05	0.65*
	δ/β	0.68*	0.67*	0.63*	0.64*	0.6*	0.61*	0.58*	0.55*	0.67*	0.62*	0.59*	0.62 ± 0.04	0.63*
	Chance	0.15 ± 0.02	0.19 ± 0.01	0.13 ± 0.02	0.16 ± 0.02	0.38 ± 0.01	0.27 ± 0.01	0.24 ± 0.01	0.21 ± 0.01	0.32 ± 0.01	0.12 ± 0.01	0.14 ± 0.02	0.21 ± 0.08	0.25 ± 0.01
REST2	δ/α	0.74*	0.76*	0.73*	0.69*	0.72*	0.61*	0.58*	0.7*	0.73*	0.76*	0.7*	0.7 ± 0.06	0.73*
	δ/β	0.78*	0.77*	0.76*	0.7*	0.74*	0.66*	0.59*	0.71*	0.69*	0.71*	0.73*	0.71 ± 0.05	0.71*
	Chance	0.11 ± 0.03	0.26 ± 0.01	0.21 ± 0.01	0.22 ± 0.01	0.35 ± 0.01	0.22 ± 0.01	0.39 ± 0.01	0.14 ± 0.02	0.13 ± 0.02	0.15 ± 0.01	0.17 ± 0.01	0.21 ± 0.09	0.21 ± 0.01
**Shallow**		**1**	**2**	**3**	**4**	**5**	**6**	**7**	**8**	**9**	**10**	**11**	**Ave**	**Mix**
MBSR1	δ/α	0.82*	0.87*	0.86*	0.83*	0.81*	0.78*	0.87*	0.79*	0.84*	0.8*	0.82*	0.82 ± 0.03	0.85*
	δ/β	0.87*	0.85*	0.88*	0.84*	0.87*	0.8*	0.85*	0.8*	0.78*	0.82*	0.81*	0.83 ± 0.03	0.83*
	Chance	0.35 ± 0.01	0.12 ± 0.02	0.37 ± 0.01	0.33 ± 0.01	0.31 ± 0.01	0.14 ± 0.02	0.32 ± 0.01	0.35 ± 0.01	0.37 ± 0.01	0.17 ± 0.03	0.35 ± 0.01	0.29 ± 0.09	0.28 ± 0.01
MBSR2	δ/α	0.97*	0.97*	0.98*	0.97*	0.95*	0.89*	0.97*	0.96*	0.97*	0.95*	0.98*	0.96 ± 0.03	0.94*
	δ/β	0.98*	0.97*	0.97*	0.98*	0.96*	0.9*	0.96*	0.95*	0.95*	0.97*	0.98*	0.96 ± 0.02	0.95*
	Chance	0.24 ± 0.01	0.29 ± 0.01	0.31 ± 0.01	0.32 ± 0.01	0.2 ± 0.01	0.17 ± 0.02	0.19 ± 0.01	0.29 ± 0.01	0.32 ± 0.01	0.35 ± 0.01	0.34 ± 0.01	0.27 ± 0.06	0.26 ± 0.01
REST1	δ/α	0.63*	0.69*	0.62*	0.69*	0.69*	0.66*	0.71*	0.68*	0.67*	0.7*	0.67*	0.67 ± 0.03	0.65*
	δ/β	0.67*	0.64*	0.69*	0.63*	0.71*	0.67*	0.68*	0.65*	0.68*	0.68*	0.66*	0.67 ± 0.02	0.64*
	Chance	0.34 ± 0.01	0.12 ± 0.03	0.35 ± 0.01	0.37 ± 0.01	0.22 ± 0.01	0.11 ± 0.01	0.32 ± 0.01	0.24 ± 0.01	0.26 ± 0.01	0.32 ± 0.01	0.32 ± 0.01	0.27 ± 0.09	0.21 ± 0.01
REST2	δ/α	0.77*	0.76*	0.79*	0.77*	0.79*	0.75*	0.8*	0.77*	0.73*	0.77*	0.71*	0.76 ± 0.03	0.74*
	δ/β	0.78*	0.74*	0.71*	0.73*	0.82*	0.77*	0.74*	0.78*	0.79*	0.72*	0.75*	0.76 ± 0.03	0.78*
	Chance	0.31 ± 0.01	0.21 ± 0.01	0.36 ± 0.01	0.28 ± 0.01	0.17 ± 0.01	0.18 ± 0.02	0.19 ± 0.01	0.33 ± 0.01	0.35 ± 0.01	0.32 ± 0.01	0.35 ± 0.01	0.28 ± 0.07	0.22 ± 0.01

As we can see from Table 7, after the CCA analysis, the *r*-value between CNN-based features and the handcraft-based features shows a high correlation (*r*-value ranging from 0.54 to 0.99) and significance (*p* < 0.05), and they are all higher than the chance level, demonstrating the features learned by the CNN models are correlative to mindfulness-related features in EEG. Besides, the *r*-values between the CNN-based features and the handcraft-based feature at MBSR1 are around 0.82, the *r*-values between the CNN-based features and the handcraft-based feature at MBSR2 are around 0.95, the *r*-values between the CNN-based features and the handcraft-based feature at REST1 are around 0.62, the *r*-values between the CNN-based features and the handcraft-based feature at REST2 are around 0.72, showing that the features learned by the CNN models are MBSR tasks related features.

## 5. Limitations and future research

This study has some noticeable limitations. First, the study is based on a small EEG dataset of only 11 non-homogeneous subjects covering both Asians and Caucasians, resulting great inter-subject variability which is difficult to be sufficiently represented by both deep learning and traditional machine learning methods. Future studies should collect EEG data from a large number of subjects participating MBSR training with no meditation experience, and this may require a multi-center collaboration. Second, in this study for the first time we perform recognition of both state and trait effect of short term MBSR training through investigating the EEG measurements at two stages of early and late training, but only a single session of EEG measurement is performed for each stage, which limits us to investigate inter-session variability and distinguish it from trait effect of the MBSR training. In future research multi-session EEG data should be collected and utilized for classification of resting and meditation states at different stages. Finally, due to the availability of only a small dataset, we use only a very simple deep learning network of shallow ConvNet and its modification with a slightly deeper architecture, deep ConvNet. Future studies may consider more complex deep learning architectures which can cope with small data and inter-subject variability in various ways. We may use a GAN structure for intrinsic data augmentation (Fu et al., 2022), use i-vector to reduce the effect of inter-subject variability for inter-subject classification (Xu et al., 2022), and use large amount of publicly available meditation independent EEG data for self-supervised learning (SSL) to assist deep learning with small data and inter-subject variability (Rafiei et al., 2022). SSL has been demonstrated effective in speech analysis using the wave2vec (Baevski et al., 2020) architecture, which has been recently adapted as neuro2vec (Wu et al., 2022) and eeg2vec (Bethge et al., 2022) for EEG signal analysis.

## 6. Conclusion and implications

This study has examined the state and trait (stage) effect of short term MBSR training using CNN based deep learning methods of deep and shallow ConvNets as well as traditional machine learning methods of CSP + SVM and FBCSP + SVM, through investigating the EEG measurements of eleven MBSR practitioners during resting and meditation at early and late training stages, supporting previous findings that short-term meditation training has EEG-recognizable state (Ahani et al., 2014) and trait (Sharma et al., 2019) effects. The classifiers are trained and evaluated using inter-subject, mix-subject, intra-subject, and subject-transfer classification strategies, each according to a specific application scenario. Results show that in the intra-subject and mix-subject classification scenarios, our deep learning classifiers have classification performance superior to related EEG-based meditation state classification studies reported in the literatures for state effect classification as well as trait effect classification using EEG data during either resting or meditation, for both novice and expert meditators with a variety of meditation types including yoga, Tibetan, and mindfulness, whereas comparing to the literatures for inter-subject classification the performance of FBCSP for novice MBSR meditators is superior for state effect recognition of novice meditators and slightly inferior but still comparable for both state and trait effects recognition of expert meditators.

Studies on clinical interventions using mindfulness meditation suggest that MBSR may reduce various mental disorders such as anxiety (Beauchemin et al., 2008), depression (Hofmann et al., 2010), ADHD (Zylowska et al., 2008), and social disorder (Beauchemin et al., 2008). EEG-based mindfulness meditation state classification can serve as a quantitative evaluation of MBSR training effectiveness and level/expertise of mindfulness of the practitioner, and then can be used as online or offline feedback to assist the practitioners for improved training and practicing performance. Our study demonstrates excellent mix-subject and intra-subject classification performance as well as reasonably good inter-subject classification. When our mindfulness state classification methods are integrated with wearable EEG sensors (Anwar et al., 2018; Álvarez Casado et al., 2021) and virtual reality (Mistry et al., 2020; Viczko et al., 2021) in future research, a neurofeedback assisted MBSR training system may be developed and can help make MBSR more effective and accessible to the general populations.

## Data availability statement

The original contributions presented in the study are included in the article/supplementary material, further inquiries can be directed to the corresponding authors.

## Ethics statement

The studies involving human participants were reviewed and approved by the Hong Kong Local Institutional Review Board (IRB). The patients/participants provided their written informed consent to participate in this study.

## Author contributions

CC, BS, FD, and XM conceptualized the project. CC and XM supervised the project. BS and RF performed data analysis. FD performed data pre-processing, results interpretation, and literature review. CC designed the methodology. JG and HS designed the experiments and collected the data. FD and CC drafted the manuscript. RF and CC revised the manuscript. All authors contributed to the article and approved the submitted version.
